# Lack of Evidence for Human-to-Human Transmission of Avian Influenza A (H9N2) Viruses in Hong Kong, China 1999^1^

**DOI:** 10.3201/eid0802.010148

**Published:** 2002-02

**Authors:** Timothy M. Uyeki, Yu-Hoi Chong, Jacqueline M. Katz, Wilina Lim, Yuk-Yin Ho, Sophia S. Wang, Thomas H.F. Tsang, Winnie Wan-Yee Au, Shuk-Chi Chan, Thomas Rowe, Jean Hu-Primmer, Jensa C. Bell, William W. Thompson, Carolyn Buxton Bridges, Nancy J. Cox, Kwok-Hang Mak, Keiji Fukuda

**Affiliations:** * Centers for Disease Control and Prevention, Atlanta, Georgia, USA; † Department of Health, Hong Kong Special Administrative Region of China

## Abstract

In April 1999, isolation of avian influenza A (H9N2) viruses from humans was confirmed for the first time. H9N2 viruses were isolated from nasopharyngeal aspirate specimens collected from two children who were hospitalized with uncomplicated, febrile, upper respiratory tract illnesses in Hong Kong during March 1999. Novel influenza viruses have the potential to initiate global pandemics if they are sufficiently transmissible among humans. We conducted four retrospective cohort studies of persons exposed to these two H9N2 patients to assess whether human-to-human transmission of avian H9N2 viruses had occurred. No serologic evidence of H9N2 infection was found in family members or health-care workers who had close contact with the H9N2-infected children, suggesting that these H9N2 viruses were not easily transmitted from person to person.

In April 1999, two World Health Organization reference laboratories independently confirmed the isolation of avian influenza A (H9N2) viruses for the first time in humans (*1*). H9N2 viruses were isolated from nasopharyngeal aspirate specimens collected from two young children who were hospitalized in Hong Kong during March 1999 (*2*). The children were not related, were hospitalized at different facilities, did not have any known contact with or link to each other, and had not traveled outside Hong Kong (*2*). Both children had uncomplicated, febrile, upper respiratory tract illnesses and fully recovered (Table 1) (*2*). Evidence for five additional human illnesses attributed to H9N2 in Guangdong Province, China, during 1998 has been reported (*3*). Detection of antibody to H9N2 has been reported from persons in northern and southern China (*3*,*4*) and poultry workers in Hong Kong (*5*), suggesting that additional unrecognized human H9N2 infections have occurred.

**Table 1:** Clinical characteristics of two children infected with influenza A (H9N2) viruses, Hong Kong, 1999^a^

Patient characteristics	History, symptoms, signs on admission	Treatment received	Laboratory studies	Clinical course	Outcome
13-month-old girl; possible failure to thrive; no recent travel	Fever 39.5°C (1 day), poor appetite, vomiting, inflamed oropharynx	Cefuroxime Paracetomol Chloropheniramine Pseudoephedrine - Triprolidine (No antiviral medications)	CRP^b^ 0.12 (mg/dL) (normal ≤0.8 mg/dL); WBC 2.22 x 10^9^; AST 66 IU/L; CXR normal; U/A normal; NP aspirate for influenza A EIA: pos NP aspirate for viral culture: pos for influenza A (H9N2), adenovirus type 3	Uneventful No fever at discharge Duration of hospitalization March 5-7, 1999	Recovered, no sequelae
4-year-old girl, mild eczema, asthma, no recent travel	Fever 38.9°C (1 day), malaise, sore throat, headache, vomiting, abdominal pain, diarrhea inflamed oropharynx	Cefuroxime Cefotaxime Beclomethosone Paracetomol (No antiviral medications)	CRP 0.25 (mg/dL); (normal ≤0.8 mg/dL); WBC 12.5 x 10^9^ (82%^N^, 10%^L^, 7%^M^); CXR: normal; blood culture neg; stool culture neg; U/A normal; NP aspirate for influenza A EIA pos; NP aspirate for viral culture pos for influenza A (H9N2)	Persistent fever, no fever at discharge. Duration of hospitalization March 1-8, 1999	Recovered, no sequelae

^a^Source: Epidemiologic investigation by the Hong Kong Department of Health and review of medical records. ^b^CRP = C-reactive protein; WBC = leukocytes; AST = aspartate aminotransferase; CXR = chest X-ray; U/A = urinalysis; NP = nasopharyngeal; EIA = enzyme immunoassay

H9N2 viruses have been prevalent in domestic poultry (chickens, ducks, geese, quail, and pigeons) throughout Asia since the early 1990s and were also isolated from swine in Hong Kong in 1998 (*6*). H9N2 viruses circulating in Asia have been classified into three antigenically and phylogenetically distinct sublineages (*7*). Two of these Asian H9N2 virus sublineages, influenza A/Quail/Hong Kong/G1/97 (G1-like lineage) and influenza A/Chicken/Hong Kong/G9/97 (G9-like lineage), were isolated from poultry in Hong Kong (*6*). The two Hong Kong children were infected by G1-like viruses, influenza A/Hong Kong/1073/99 and A/Hong Kong/1074/99 (*8*). The H9N2 viruses that have been isolated from poultry in Hong Kong are not highly pathogenic in chickens (*8*), whereas antigenic analysis of the H9N2 viruses isolated from humans in southern China suggested that they were more closely related to the G9-like viruses (*9*). However, the G1-like viruses contain internal genes that are highly homologous to those of highly pathogenic influenza A (H5N1) viruses isolated from chickens and humans in Hong Kong in 1997 (*7*).

The first and only documented human outbreak of highly pathogenic avian influenza A (H5N1) virus infections resulted in 18 hospitalizations and six deaths among Hong Kong residents during 1997 (*10*–*12*). A case-control study identified recent exposure to live poultry as an important risk factor for H5N1 infection (*13*), and cohort studies suggested that human-to-human transmission of H5N1 virus was limited (*14*,*15*). The poor transmissibility of these H5N1 viruses among humans and the elimination of approximately 1.5 million chickens appear to have been key factors that stopped this outbreak (*12*).

Avian populations, including domestic poultry and waterfowl, are the natural reservoir for all 15 known Influenza A virus (FLUAV) hemagglutinin (HA) subtypes, including H5 and H9 viruses (*16*). Viruses with novel HA can emerge when animal and human FLUAV genes undergo reassortment in the same host or when viruses from an animal host, such as swine or poultry, directly infect susceptible persons who lack protective immunity against the novel HA (*17*,*18*). In addition to ability to infect humans, the transmissibility of a novel *Influenzavirus* is a key factor influencing whether the novel virus can cause an influenza pandemic (*19*). The emergence of novel influenza A (H1N1), A (H2N2), and A (H3N2) viruses led to three influenza pandemics during the 20th century (*19*).

The identification of two children who had acute infection with novel H9N2 virus strains provided the first opportunity to investigate their transmissibility and pandemic potential among humans. We report the results of four retrospective cohort studies designed to detect serologic evidence of H9N2 virus infection among family members and health-care workers (HCWs) exposed to the two H9N2 patients, as well as unexposed controls.

## Methods

The target populations included HCWs at the two hospitals where the H9N2-infected patients received care, as well as family and household members of the patients. The infectious period for an H9N2 patient was defined as a 15-day period beginning from the day before illness onset to the 14th day after illness onset (patient 1: February 27 to March 13, 1999; patient 2: March 3 to 17, 1999). The infectious period was defined conservatively to reflect the potential for prolonged viral shedding, especially since children can shed influenza viruses for longer periods than adults. Close contact was defined as coming within 3 m of an H9N2-infected patient. Participants were defined as exposed if they had close contact with an H9N2 patient during the infectious period. An unexposed person was defined as having had no contact with the H9N2 patients during the infectious periods. Unexposed subjects included family members and relatives who did not live in the same household as and had no contact with an H9N2 patient, and HCWs who worked on hospital units different from those where the H9N2 patients were located and who denied exposure to the H9N2 patients.

### Study Design

We conducted four retrospective cohort studies of either HCWs or family and household members of the H9N2 patients. During face-to-face interviews conducted in either English or Cantonese, staff from the Hong Kong Department of Health administered a detailed questionnaire to a group of household members, family members, and relatives of each H9N2-infected child. The questionnaire assessed the level of exposure and contact with the H9N2-infected patient during the infectious period, along with other suspected risk factors for H9N2 infection, such as recent contact with poultry and swine. A similar questionnaire administered to HCWs asked about contact with each H9N2-infected patient during the patient’s hospitalizations (patient 1: March 1-8, 1999; patient 2: March 5-7, 1999), and recent exposure to poultry and swine. All participants provided written, signed informed consent. Approximately 10 cc of blood was provided by each participant approximately 5 to 6 weeks (except where indicated) after the onset of the H9N2 patients’ illnesses to test for antibody to H9N2.

### Serologic Testing

Serum samples from all study participants and the two H9N2 patients were tested for antibody to FLUAV H9N2 by a microneutralization assay at both the Centers for Disease Control and Prevention (CDC), Atlanta, and the Hong Kong Department of Health Government Virus Unit Laboratory, as described (*20*), except that A/Hong Kong/1073/99 (HK/1073; H9N2) virus, isolated from patient 1, was used in the assay. Specimens from H9N2 patients were single serum samples collected 35 days (patient 2) and 39 days (patient 1) after illness onset. The virus isolated from patient 2 (A/Hong Kong/1074/99) was antigenically indistinguishable from HK/1073. Sera were considered positive by microneutralization if anti-H9 titers ≥80 were obtained in at least two independent assays.

At CDC, a Western blot assay with bromelain-purified or baculovirus-expressed recombinant hemagglutinin (rHA; Protein Sciences, Inc., Meriden, CT) from HK/1073 virus was used to confirm each positive microneutralization result, as described (*14*). Microneutralization-positive sera were adsorbed with FLUAV H3N2 viruses to remove antibodies that were cross-reactive among FLUAV subtypes before retesting by microneutralization assay. Serum (50 µL) mixed with 100 μg of purified virus was incubated 45 min at 20°C and then 2 h at 4°C. Virus was pelleted by ultracentrifugation (30 min at 45,000 rpm and 4°C). To remove residual virus, serum was further adsorbed twice with 10% v/v turkey red blood cells (RBC) (30 min at 4°C) and then centrifuged to remove RBC (2 min at 12,000 rpm).

At the Government Virus Unit in Hong Kong, microneutralization-positive sera were confirmed by a single radial hemolysis assay for H9N2 antibodies, based on a modified, previously described protocol (*21*). HK/1073 virus-turkey RBC complexes, cross-linked by chromium, and complement were suspended in an agarose matrix. Sera were added to 2-mm diameter agarose wells. After overnight incubation at 35°C, a zone of hemolysis around the wells indicated the presence of anti-H9N2 antibodies. Sera producing hemolysis were absorbed with HK/1073 virus concentrate by mixing 15 μL of sera with 5 μL of virus concentrate, followed by a 1-h incubation at room temperature. The mixture was then retested as described. The absence of hemolysis confirmed the presence of H9N2 antibody. Absorption with A/Sydney/05/97 (H3N2)-like and A/Beijing/262/95 (H1N1)-like viruses was done to remove the nonspecific zones so only H9N2 antibody reacted on the single radial hemolysis plates. Sera were considered positive for H9N2 antibodies if the microneutralization assay and all confirmatory tests were positive in both laboratories.

Sera from the two H9N2-infected children were also tested by enzyme-linked immunosorbent assay (ELISA) to detect immunoglobulin (Ig) G and IgM antibodies to H9 as described (*14*), except that HK/1073 rHA (1 μg/mL) was used as the antigen. ELISA titers were calculated as the reciprocal of the highest dilution of sera that gave an *A*_490_ value greater than the mean *A*_490_ plus 3 standard deviations of six to seven negative age-matched controls at an equivalent dilution of sera. A titer ≥1,600 was considered positive.

### Statistical Analysis

Univariate analysis of associations between exposure variables and antibodies to H9N2 virus results were done by SAS 6.12 (SAS Institute Inc., Cary, NC).

## Results

### Serologic Response to H9N2 Virus Infection

Patient 1 was positive for H9 antibodies to H9 by all serologic assays and had substantial titers of H9 HA-specific IgG and IgM antibodies (Table 2). Low titers of H9 HA-specific IgG and IgM antibodies were detected by ELISA in serum from patient 2, but no neutralizing antibody response was detected.

**Table 2:** Serologic responses of two patients from Hong Kong infected with influenza A (H9N2) virus

Patient	Age (years)	Sex	Serologic anti-H9 response				
			Days post symptom onset	Neutralizing antibody titer^a^	Western blot^b^	ELISA IgG^c^	ELISA IgM^c^
1	4	female	39	135	Positive	51200	18100
2	1	female	35	40	Positive	6400	1600

^a^Titers expressed as the geometric mean of four replicate titers; titers ≥80 were considered positive for anti-H9 antibodies. ^b^Western blots were performed by using a purified baculovirus-expressed recombinant HK/1073 HA as antigen. ^c^Enzyme-linked immunosorbent assay (ELISA) immunoglobulin (Ig) G and IgM antibodies were detected on plates coated with purified baculovirus-expressed recombinant HK/1073 HA (1 μg/mL). Titers are expressed as the geometric mean of duplicate endpoint titers estimated as described in Methods. A titer ≥1,600 was considered positive for anti-H9 antibodies.

### Study Participants

The demographic characteristics of the study participants are shown in Table 3. For H9N2 patient 1, exposed and unexposed family members or HCWs did not differ significantly by age or sex. For H9N2 patient 2, exposed and unexposed HCWs did not differ by age or sex. For family members of H9N2 patient 2, the unexposed participant was older than the exposed participants, but the number of study participants was very small. In the HCW cohorts of both H9N2 patients, more participants were women than men.

**Table 3:** H9N2 serologic results of cohort studies involving family members and health-care workers, Hong Kong, 1999

	Patient 1		Patient 2	
Family members	Exposed (n=3)	Unexposed (n=11)	Exposed (n=6)	Unexposed (n=1)
Median age (years), range	30 (2 to 31)	31 (<1 to 39)	31.5 (2 to 55)	68
Male:female	0.043055556	1:0.8	0.042361111	0.0416666667
Seropositive	0	0	0	0
Health-care workers	Exposed (n=30)	Unexposed (n=75)	Exposed (n=15)	Unexposed (n=23)
Median age (years), range	29.5 (19 to 51)	28 (19 to 59)	36 (24 to 56)	36 (25 to 50)
Male:female	0.044444444	1:3.4	0.051388889	0.0159722222
Seropositive	0	1	0	0

### Family Member Cohort Studies

Fourteen of 15 eligible persons were enrolled in the family cohort study (3 exposed immediate family members and 11 unexposed relatives) of H9N2 patient 1. One exposed participant reported respiratory symptoms within the 2 weeks after onset of illness in the patient. This participant’s serum was obtained 3 weeks after H9N2 patient’s illness onset and was seronegative for H9N2 antibodies. No other participant reported respiratory illness. All 14 study participants tested seronegative for H9N2 antibodies (Table 3).

All seven family and household members eligible for the family cohort study of H9N2 patient 2 were enrolled (six exposed and one unexposed family and household members). Two exposed participants reported respiratory symptoms within 2 weeks after onset of illness in H9N2 patient 2. The unexposed participant reported no respiratory illness. All seven study participants tested seronegative for H9N2 antibodies (Table 3).

### HCW Cohort Studies

The HCW study population for H9N2 patient 1 consisted of 30 exposed HCWs from 4 hospital units and 75 unexposed HCWs from 14 hospital units. Three exposed and three unexposed HCWs reported respiratory symptoms (cough, sore throat, or rhinorrhea) during H9N2 patient 1’s hospitalization or within 5 days of the date of hospital discharge. All 30 exposed study participants were seronegative for H9N2 antibodies. One of the 75 unexposed HCWs was seropositive (Table 3). The HCW who tested seropositive for antibodies to H9N2 had no known exposure to a confirmed H9N2-infected patient and reported no contact with poultry or swine.

The HCW study population for H9N2 patient 2 was 15 exposed and 23 unexposed HCWs from four hospital units. One exposed HCW declined to participate. Four exposed HCWs reported respiratory symptoms beginning 2 to 5 weeks after contact with the patient. All 38 study participants tested seronegative for H9N2 antibodies.

## Discussion

These cohort studies suggest that influenza A (H9N2) viruses were not transmitted from the two H9N2-infected children to family and household members or HCWs who were exposed to the H9N2 patients during their acute illness infectious periods. As described for the avian influenza A (H5N1) viruses (*15*,*20*), a combination of serologic assays was effective in detecting H9 virus-specific antibodies in two pediatric cases of H9N2 infection. However, the same serologic assays did not detect H9 antibodies in family members or HCWs exposed to the H9N2 patients. Only two known exposed persons, an HCW and a family member of one H9N2 patient, declined to participate in the studies. The HCW who tested seropositive for antibodies to H9N2 had no known exposure to a patient with confirmed H9N2 infection or contact with poultry or swine. The timing of H9N2 infection in this HCW could not be determined.

Evidence for influenza A (H9N2) infection as the cause of acute illness in the two patients includes the direct isolation of H9N2 viruses from nasopharyngeal aspirate specimens during the acute phase of illness (*1*) and the detection of H9-specific IgM antibodies, suggesting recent infection with an H9 virus. No other bacterial or viral pathogens were identified except for isolation of adenovirus type 3 from patient 1. The significance of the latter finding is unknown since this patient did not have typical signs of adenovirus type 3 infection, such as conjunctivitis. Isolation of adenovirus in this patient could represent acute atypical infection, acute subclinical infection, or persistent viral shedding from previous adenovirus infection.

The apparent lack of human-to-human transmission of avian H9N2 viruses and the low transmissibility of avian H5N1 viruses among humans have several possible explanations (*14*,*15*). The genomes of the H9N2 and H5N1 strains that were isolated from humans were derived entirely from avian influenza viruses; no reassortment with circulating human influenza A viruses had occurred. It is possible that the avian virus genome limits viral spread among humans. The molecular basis of influenza virus transmission among humans and other species remains poorly understood. However, following the introduction of an avian virus into humans, alterations in receptor-binding specificity of the HA are likely necessary for effective human-to-human transmission (*22*). Alternatively, the children may not have shed H9N2 virus in titers sufficient to facilitate transmission to other persons. Neither H9N2-infected child had coughing or sneezing that would have enhanced transmission to persons who had close contact with them.

To improve specificity for detecting antibody for H9N2 over that of the hemagglutination-inhibition antibody assays used previously (*3*), we used a combination of confirmatory tests and an adsorption step to reduce cross-reactivity with antibodies to other influenza viruses. Sera testing positive by neutralization test were then tested by Western blot assay. Sera positive for both these assays were further tested by neutralization assay following adsorption of sera with influenza A (H3N2) viruses. Sera that were negative for antibodies to H9N2 by neutralization assay were not tested by Western blot because of resource limitations. However, all sera from children who were contacts of the H9N2 patients, as well as the patients themselves, were also tested by an H9-specific ELISA. Both patients but none of the exposed children tested positive for H9 antibodies.

Because of insufficient sera, the H9N2 patients were not tested for antibodies to neuraminidase (NA). The N2 NA of the H9N2 viruses isolated from patients is antigenically distinct from that of recent H3N2 human viruses, although some cross-reactivity with human H2N2 and early H3N2 viruses has been reported (*8*). However, additional studies from our laboratory indicate that the apparent cross-reactive antibodies that could be removed from some human sera by adsorption with H3N2 viruses was not due to cross-reactivity between the N2 NAs, since these sera also reacted with a reassortment H9N7 virus (CDC, unpub. data).

Because only two H9N2 cases were identified, we did not conduct a case-control study to identify risk factors for H9N2 infection. Thus, the sources and modes of acquisition of H9N2 for the two infected children are unknown. The Hong Kong Department of Health found that one H9N2 patient had very brief exposure to live chickens 11 days before onset of illness but did not directly touch the birds. No other contacts with live poultry, swine, or other animals for either H9N2 patient were found. There was no known contact or common exposure between the two H9N2 patients.

During the 1997 FLUAV (H5N1) outbreak in Hong Kong, a case-control study found that visiting a poultry stall or market with live poultry during the week preceding illness was the main risk factor for H5N1 infection (*12*). During that outbreak, the Hong Kong Department of Health enhanced its active surveillance for influenza-like illness and influenza viruses in hospitals, general outpatient clinics, and physicians’ offices. This enhanced surveillance system detected the two novel H9N2 infections.

We were able to obtain only one convalescent-phase blood specimen from study participants, which limited our ability to document seroconversion. However, none of the exposed persons were seropositive for H9N2. Currently, there are no seroprevalence data on rates of H9N2 infection in children or the general population. One study of a cohort of poultry workers in Hong Kong found that approximately 30% were seropositive for antibodies to H9N2 (*5*). Ongoing surveillance and availability of H9N2-specific reagents should facilitate timely identification of H9N2 infection and allow collection of paired sera for further studies of person-to-person transmission.

In addition to H9N2, other avian influenza viruses have been isolated from specimens collected from Hong Kong poultry since 1997, including H6, H4, and H11 viruses (*23*). During April and May 2001, highly pathogenic avian influenza A (H5N1) viruses were again isolated from live poultry in Hong Kong markets (*24*). After chicken deaths were observed in some markets, the Hong Kong government temporarily closed all wholesale and retail live poultry markets for cleaning, stopped importing poultry from China, and slaughtered approximately 1.3 million birds during May 2001. The poultry markets reopened in June 2001. No human illnesses attributed to avian influenza viruses have been identified since the two H9N2 cases in 1999. However, these recent events have heightened the need to understand the public health risk of H5N1, H9N2, and other avian influenza viruses.

These limited studies suggest that avian influenza A (H9N2) viruses were not transmitted from the two infected children to exposed household members, relatives, or HCWs in Hong Kong. However, H9N2 viruses are widely distributed in avian populations, can infect humans, and could evolve or undergo genetic reassortment with potential for increased pathogenicity and transmissibility in humans. The recent emergence of human infections with avian influenza A (H9N2) and (H5N1) viruses highlights the need to improve surveillance for influenza viruses in poultry, swine, and humans, especially in Asia. Further studies to assess the health risks posed by H9N2 and other avian influenza viruses are warranted.
